# Fevers

**Published:** 1905-08-26

**Authors:** 


### Progress in Medicine and Surgery.

### FEVERS.

**The Prevention of Yellow Fever.**—The discovery that yellow fever is due to the bite of an infected mosquito has produced far-reaching results, not only to health, but also to commerce and trade in regions where this much-dreaded pest prevails. Although by some the mosquito has not yet been accepted as the only source of infection, overwhelming evidence continues to be forthcoming that the disease is effectually checked in its progress where the only preventive measures employed are those directed against the mosquito. Dr. A. H. Doty,[1] Health Officer of the Port of New York, has recently again drawn attention to the subject. He states that exhaustive experiments have been made at the New York Quarantine Station proving conclusively that mosquitos cannot live in closed receptacles, such as bags, trunks, etc., for more than 24 or 30 hours, and that therefore the disinfection of baggage which has been in transit more than two days—and which has not, presumably, been disturbed during that time—is unnecessary. In a paper recently read at the fourth Pan-American Medical Congress,[2] the subject is also dealt with by Dr. Finlay, of Cuba, who once more emphasises the point that to keep yellow fever patients from being bitten by mosquitos of the genus *Stegomyia fasciata* is the only means of arresting the spread of the disease. This is readily effected by confining all patients in mosquito-proof chambers and destroying by fumigation with pyrethrum or sulphur any mosquitos found. Early diagnosis is also obviously an essential to complete treatment. In Havana no case has occurred for three years, and similar good results have been obtained in Vera Cruz, in Sao Paolo, and in Rio Janeiro, by the employment of these means alone. The relief to commerce obtained by these discoveries and observations is, of course, immense, as, in the old days of belief in infection by personal contact and fomites of all descriptions, the quarantine laws enforced practically paralysed trade in affected quarters during an epidemic. Dr. Purnell and others point to epidemics in St. Thomas and elsewhere which were not, in their belief, traceable to mosquito infection, and which were stamped out by cleanliness; and a belief in the infectivity of fomites still prevails in the Mississippi valley. But mosquitos have probably shared destruction with other vermin in cleansing processes, and, to take one example, in Havana, where adherence to the mosquito theory has stamped out the disease, the worst epidemic known for 18 years occurred directly after the city had been "cleaned clean."

**Cholera at Manila.**—The epidemic of Asiatic cholera which has recently raged in the Philippine Islands, and which has now subsided, has stimulated investigation into the nature and treatment of the disease. Haffkine's protective inoculations were proved to be impracticable among the Filipinos, but the experimental work of R. P. Strong[3] has produced some practical results. By the autolytic digestion of carefully killed cholera spirilla in an aqueous fluid the "receptors" were found to become separated from the bacterial cells, so that they could be filtered off in solution. The solution of free receptors injected into man or animals is said to produce a blood serum which, though it has little antitoxic power, possesses high bactericidal and agglutinative properties. The injection appears to be free from danger and attended by very little local disturbance, and hence there is hope of its usefulness as a prophylactic serum for man. In Manila the epidemic appeared to be largely spread by food contamination, but ordinary hygienic measures directed to food and water supplies were inadequate for eradicating the disease. The discovery of a safe and efficient prophylactic serum would be of very great value.

**Scarlet Fever.**—The treatment of scarlet fever by means of antistreptococcic serum is receiving attention. A streptococcus is generally admitted to be found more constantly than any other organism in this disease, although its *rôle*, whether that of primary cause or secondary infection, is not yet fully determined.[4] Moser has isolated nearly 30 different strains of streptococcus, and by injecting all these into the horse he has obtained a serum of corresponding multivalency. By injecting this polyvalent serum, which is not yet on the market, into a series of very severe cases in large doses of 200 c.c. considerable reduction of mortality has been obtained and complications and sequelæ have been diminished. Sickness and a rash may follow its use, but in favourable cases pulse and temperature fall rapidly without sweating or collapse, and the nervous symptoms are ameliorated rapidly. A multivalent serum has also been employed by Dr. J. H. Marsh[5] in six highly toxic cases occurring at Macclesfield in children under eight. Three of the patients recovered—a more favourable result than would usually follow in cases of such severity. The serum employed was one prepared by Messrs. Burroughs and Wellcome by immunising horses against ten varieties of streptococcus. Smaller doses were employed than Moser uses—10 c.c. repeated every four hours—and antiseptic nasal and throat douches and cold sponging were also used. Though the number of cases treated is too small for generalisation, the results are hopeful. Turning from its treatment to other aspects of the scarlet fever question, there is a noticeable inclination to question or distrust the value of hospital isolation. Dr. Bliss[6] points out that the low mortality which has coincided with the period of hospital isolation is more probably due to the mild type of the disease which appears to prevail in most countries at present, while cases treated in the Metropolitan Asylums Board hospitals give, if anything, a slightly higher death-rate than home-treated cases. This argument loses sight of the fact that probably a larger number of severe cases would be sent into the hospitals. Dr. Killick Millard also finds no diminution in mortality or average attack-rate since the formation of isolation hospitals. Early and accurate diagnosis is an important factor in rendering isolation effective, and that is often difficult or impossible. A cutaneous reaction has been observed by G. A. Ferraby,[7] which may help to this end. He has noticed that a brilliant white line follows in about 15 seconds the pressure of a smooth blunt instrument over most erythematous surfaces, but that in scarlet fever this is but very feebly marked.

[1] Med. Rec., Feb. 11, p. 237. [2] Ibid., p. 233. [3] Ibid., Feb. 18. [4] Edin. Med. Jour., Feb., p. 202. [5] Brit. Med. Jour., Feb. 18, p. 355. [6] Med. Chron., March, p. 386. [7] Brit. Med. Jour., Feb. 18, p. 355.

(*To be continued.*)

